# Ex Ovo Chorioallantoic Membrane Assay as a Model of Bone Formation by Biomaterials

**DOI:** 10.1021/acsmacrolett.4c00343

**Published:** 2024-09-26

**Authors:** Nazanin Owji, Nupur Kohli, Oliver G Frost, Prasad Sawadkar, Martyn Snow, Jonathan C Knowles, Elena García-Gareta

**Affiliations:** † Regenerative Biomaterials Research Group, The RAFT Institute and The Griffin Institute, Northwick Park and Saint Mark’s Hospitals, Harrow HA1 3UJ, United Kingdom; ‡ Division of Biomaterials and Tissue Engineering, Eastman Dental Institute, University College London, London NW3 2QG, United Kingdom; § Department of Biochemical Engineering, University College London, London WC1E 6BT, United Kingdom; ∥ Department of Biomedical Engineering, Khalifa University of Science and Technology, Abu Dhabi 127788, United Arab Emirates; ⊥ Healthcare Engineering Innovation Center, Khalifa University of Science and Technology, Abu Dhabi 127788, United Arab Emirates; # Royal Orthopaedic Hospital NHS Foundation Trust, Birmingham B31 2AP, United Kingdom; ○ Multiscale in Mechanical and Biological Engineering Research Group, Aragon Institute of Engineering Research (I3A), University of Zaragoza, Zaragoza 50018, Aragon, Spain; □ Aragon Institute of Healthcare Research (IIS Aragon), Miguel Servet University Hospital, Zaragoza 50009, Aragon, Spain

## Abstract

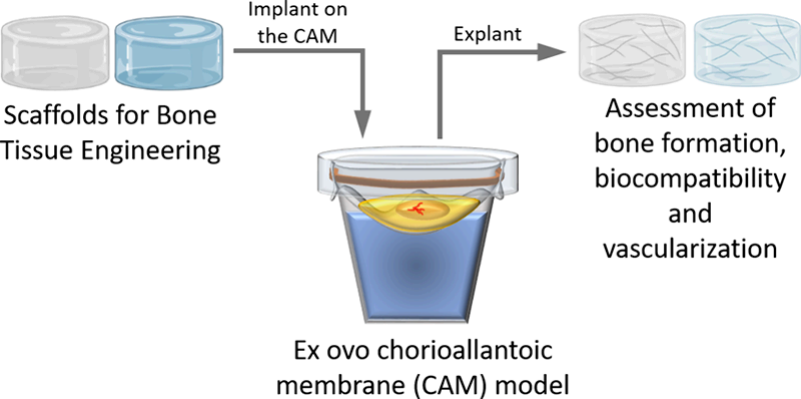

Biomaterials play an increasingly critical role in bone tissue engineering. However, achieving effective clinical translation requires a careful choice of biomimetic materials and thorough assessment of their efficacy and safety. Existing *in vitro* and *in vivo* models have drawbacks including time and cost constraints, invasive procedures, and discordance between animal models and clinical outcomes. Therefore, there is a demand for an alternative model. We hypothesized that the chick embryo chorioallantoic membrane can serve as a bioreactor to evaluate the initial sign of bone formation on scaffolds. In parallel, we investigated the osteogenic potential of a previously fabricated fibrin-alginate-calcium phosphate biomaterial (FACaP). Blood vessels were observed to infiltrate the scaffolds with early signs of bone formation, confirmed via RUNX-2 and alpha smooth muscle actin markers. The scaffolds’ chemical composition was evaluated by Fourier-transform infrared spectroscopy, and ion chromatography was used to assess calcium ion release. Finally, the topography was examined by atomic force microscopy. In conclusion, this system offers simple refinement for *in vivo* models in bone tissue engineering and highlights the great potential of FACaP as an angiogenic and osteogenic biomaterial for non-load-bearing applications.

Bone defects are one of the leading causes of disability and a major socio-economic burden: worldwide, an estimated 2.2 million bone graft procedures are performed annually.^1,2^ The “gold standard” autograft presents a limited supply and donor site morbidity. Alternative allografts display a low-grade immune reaction and high costs.^3^ Therefore, there is a need for effective bone scaffolds.^4^ However, poor vascularization, a contributing factor in impaired bone healing, remains a central challenge.^5^ Thus, there is a need to design and develop scaffolds that promote vascularization and aid bone repair by using specific minerals, cells, and growth factors coupled with a mechanically stable material.^6,7^ However, regulatory approval for clinical use requires thorough evaluation.^8^ Initially, this entails the assessment of cytotoxicity, cell–material interaction, and scaffold functionality. Due to the intricate nature of biological systems, including blood supply, immune response, and interactions among various cell types, it is crucial to employ animal models before clinical assessments.^9^ However, experimental inconsistencies and variables, such as animal age, physiology, and bone composition, have led to unreproducible standard animal models in bone tissue engineering.^10^ Such limitations, combined with the ethical obligations to reduce, refine, and replace (3Rs) animal usage in research^11^ highlight the importance of developing new models that allow more accurate recapitulation of a dynamic *in vivo* environment.

An approach is using the chorioallantoic membrane (CAM) assay, where a material is implanted onto the extracellular membrane of a developing chicken embryo. The CAM assay has found extensive applications in the study of angiogenesis, tumor cell invasion, and metastasis.^12−14^ In tissue engineering, it serves as an indicator of biocompatibility and angiogenic response and acts as an intermediary step between *in vitro* and *in vivo* models.^14^ The highly vascularized CAM significantly enhances the efficiency of interactions with biomaterials, providing advantages such as high reproducibility, simplicity, and cost-effectiveness.^13^ Importantly, the CAM is minimally invasive and causes no pain to the embryo, as it lacks innervation. Therefore, it serves as a model that represents a refinement in terms of animal welfare. In the present work, we aimed to expand the use of the CAM assay into bone tissue engineering research by using it to assess early bone formation by bone biomaterials.

In this study, we first tested the hypothesis that the CAM model can function as a living bioreactor to assess *in vivo* bone formation by biomaterials. Second, we investigated the osteogenic potential of a pro-angiogenic, porous, cross-linked, and biodegradable fibrin/alginate scaffold with deposits of calcium phosphate (CaP) that was originally developed for wound healing and later adapted for bone tissue engineering.^15−18^

Biomaterials used in this study are described in Table 1 and Figure 1. The demineralized bone matrix (DBM) is clinically available, and the fibrin/alginate (FA) material was developed in our laboratory.^15,18^ FACaP composites were prepared by immersing the FA material in concentrated simulated body fluid (SBF) solutions, yielding two prototypes: FACaP1 and FACaP2.^17^

**Figure 1:**
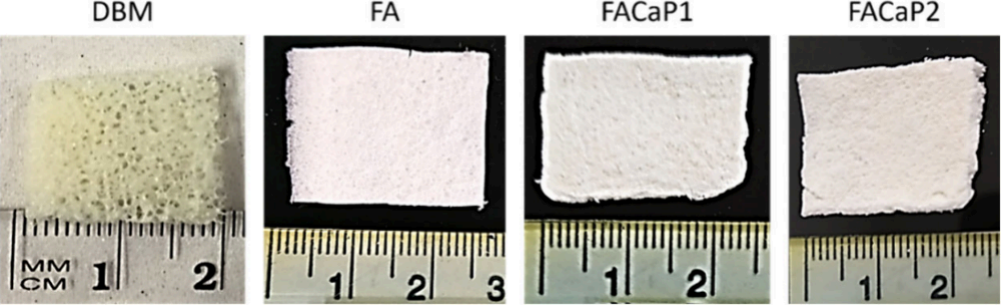
Macroscopic appearance of biomaterials.

**Table 1:** Description of Biomaterials^15−18^

biomaterial	description	% porosity (mean ± SD)
demineralized bone matrix (DBM)	3D matrix of collagen I and noncollagenous proteins present in the bone ECM.	62.2 ± 4.4
fibrin/alginate (FA)	3D cross-linked porous matrix of bovine fibrin and alginate.	93.8 ± 3.5
fibrin/alginate-CaP 1 (FACaP1)	3D cross-linked porous matrix of bovine fibrin and alginate coated throughout with mineral deposits of amorphous CaP phases containing Ca, P and Mg.	92.9 ± 0.2
fibrin/alginate-CaP 2 (FACaP2)	3D cross-linked porous matrix of bovine fibrin and alginate coated throughout with mineral deposits of octacalcium phosphate (OCP) and hydroxyapatite (HA).	88.9 ± 1.9

DBM presented a yellowish color while FA and FACaP were white (Figure 1). They all appeared as porous meshes that were easily handled by hand and forceps and cut using a scalpel. Handleability and aesthetics suggest possible applications of FACaP as bone filler in dentistry.

Scanning electron microscopy (SEM; Figure 2) showed that both DBM and FA presented a complex porous mesh of fibers in the micro- and nanoscale, which is important in enhancing cell attachment, resulting in bioactivity and biocompatibility.^15^ FACaP1 and FACaP2 displayed mineral deposits: while in FACaP1 they were globular and amorphous and had micro- and nanosizes, and in FACaP2 they had a plate-like morphology in the microscale arranging in larger cauliflower-like structures. These morphological differences can influence the osteogenic and angiogenic properties of each scaffold.^16,17,19^

**Figure 2:**
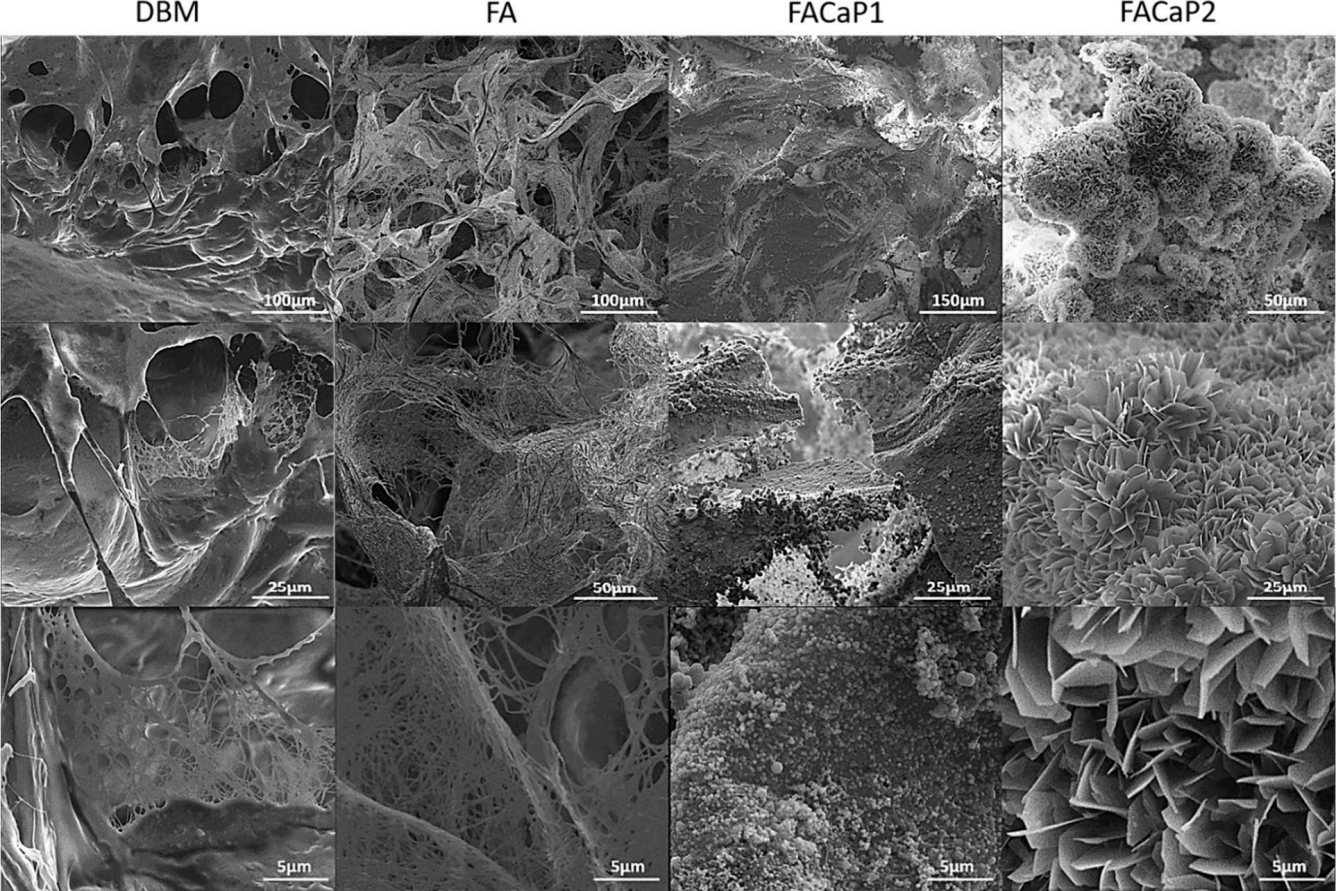
SEM images of the biomaterials.

Figure 3 graphically summarizes the timeline of the *ex ovo* CAM assay of this study, which was developed in our laboratory and does not require ethical approval.^16^ On embryonic day 0 (ED0), fertile chicken eggs were incubated at 38 °C and 45–50% humidity until ED3, when they were transferred to a glass culture setup. The chick embryo and CAM developed over time until a clearly visible network of blood vessels surrounded the embryo on ED9, when materials (5 × 5 mm in size) were implanted. On ED12, materials were explanted and photographed by stereomicroscopy. Afterward, samples were processed for immunohistochemistry with RUNX-2, α-smooth muscle actin (αSMA), and DAPI. The fluorescence images were acquired by confocal laser scanning microscopy.

**Figure 3:**
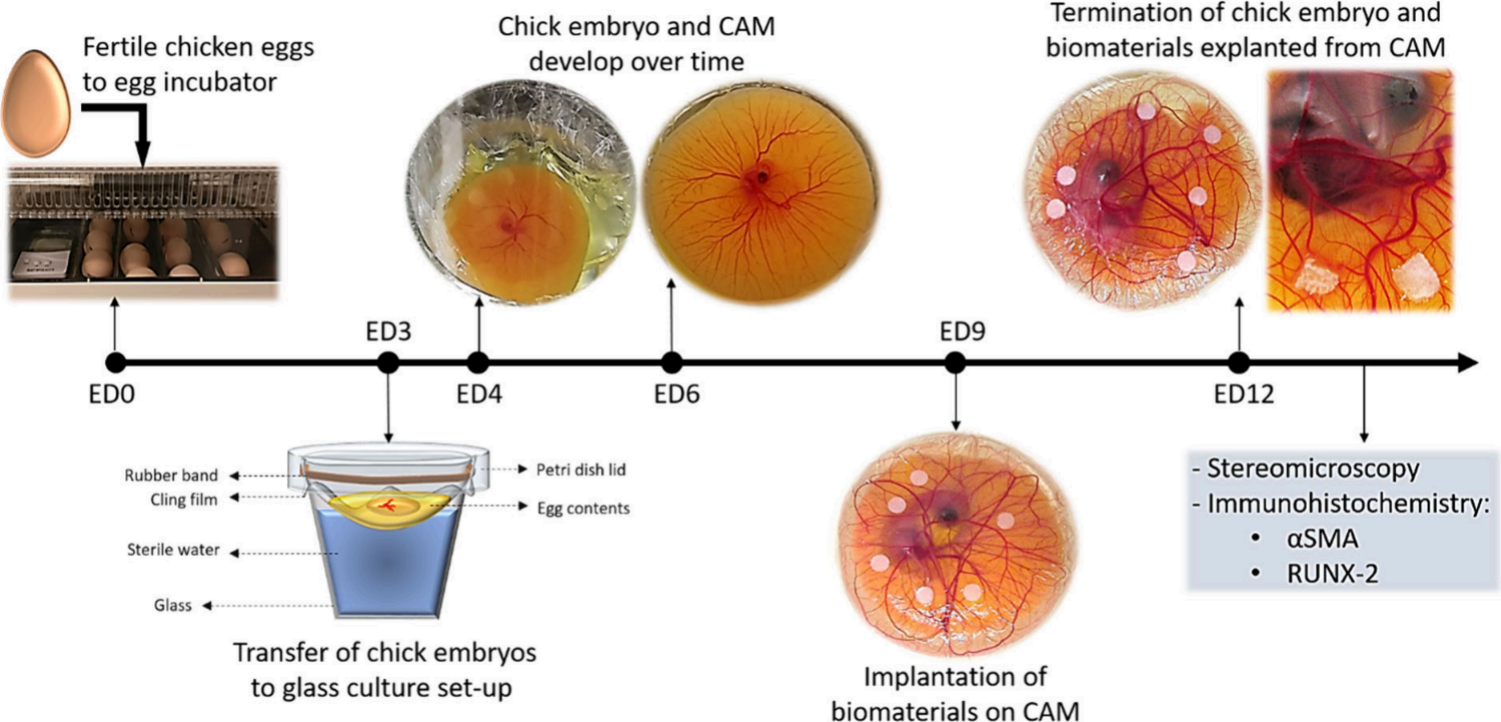
Timeline of the *ex ovo* CAM assay and post-assay analysis. The egg incubator image and the glass culture setup scheme were already published in Kohli et al. 2020^16^ (open access article distributed under the terms and conditions of the Creative Commons Attribution (CC BY) license).

The CAM assay showed the angiogenic capacity and biocompatibility of the scaffolds (Figure 4), confirming the angiogenic potential of FACaP1 and FACaP2, where blood vessels were seen to infiltrate them from the periphery all the way to the middle (Figure 4A). The percentage of the vascular area showed no significant differences (Figure 4B). The greatest percentage was shown by FA, which mostly comprises fibrin, a well-known pro-angiogenic biopolymer. The number of bifurcation points in FACaP1 was significantly higher than in FACaP2. Angiogenesis is viewed as fundamental in the regeneration of different tissues.^20−22^ Additionally, native bone is a significantly vascularized tissue, hence, it is essential to fabricate scaffolds that can recapitulate this. The unique composition of FACaP1 and FACaP2 with adequate porosity allowed vascular infiltration. In particular, FACaP1 showed increased vessel sprouting potential, which could be due to the morphology of the CaP deposited on this material leading to easier dissolution and the subsequent release of calcium ions that can stimulate angiogenesis driven by cellular phenotypic changes.^17^

**Figure 4:**
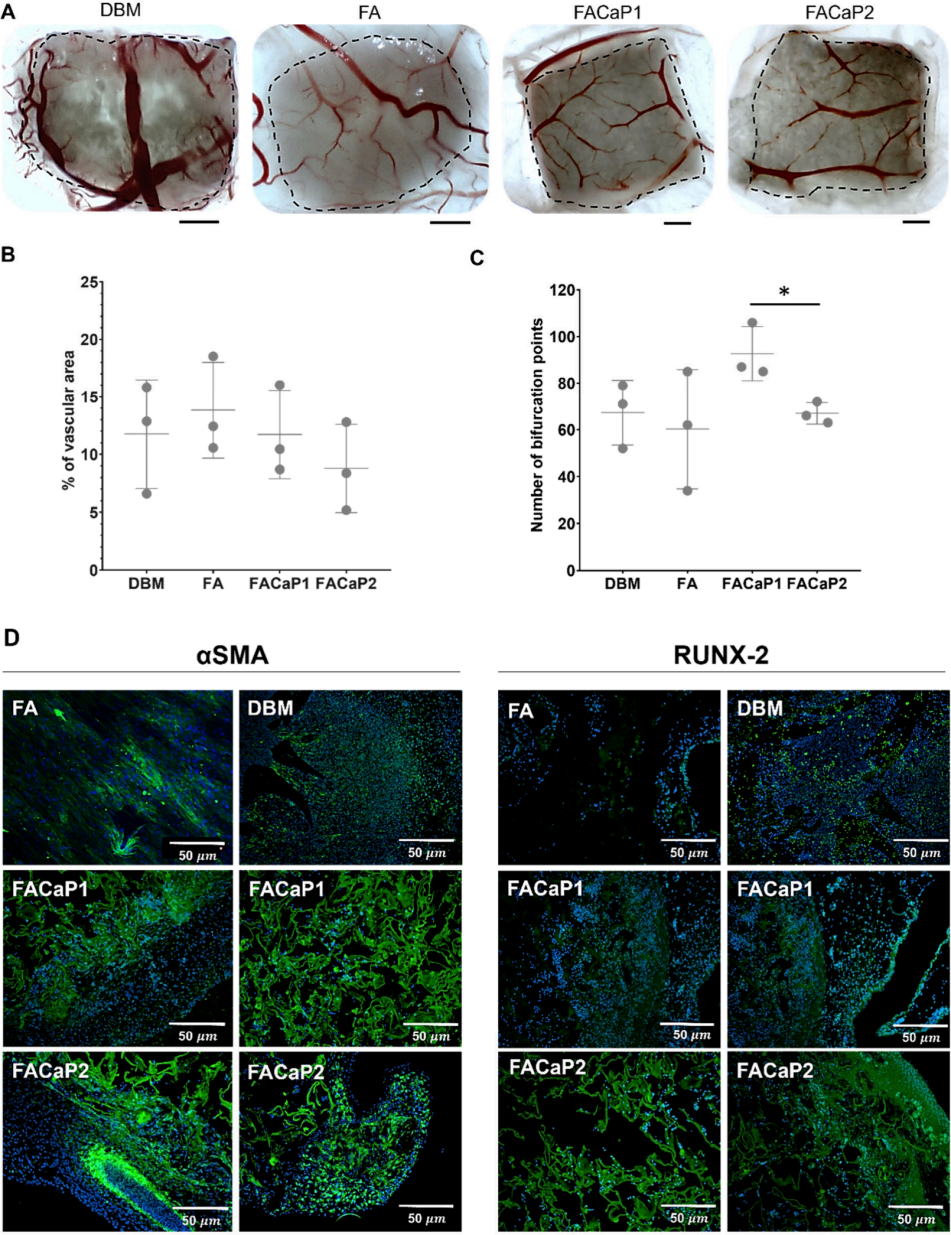
A) Stereomicroscopic images of scaffolds (demarked by the dotted line) on the CAM. Scale bar = 1 mm. (B) Percentage vascular area. (C) Number of bifurcation points. Graphs show mean ± SD of *N* = 3 (individual values shown). *p < 0.05. (D) Representative images of immunohistochemistry. Green: expression of αSMA (left) or RUNX-2 (right). Blue is cell nuclei (DAPI stained).

Immunostaining of αSMA, an actin protein in vessel walls, verified signs of blood vessel formation across all samples (Figure 4D). Qualitatively, greater αSMA expression was observed in FACaP1 compared with FACaP2, which correlates with the results in Figure 4B,C. This may be explained by the globular and amorphous morphology of CaP in FACaP1, suggesting that it would rapidly dissolve, thereby releasing Ca^2+^ ions, which have been shown to promote angiogenesis.^17^

To assess the *ex ovo* CAM model as a bioreactor for the investigation of osteogenic potential of biomaterials, RUNX-2 was looked at, since it is a master regulator of bone development and the furthest upstream transcription factor in the regulation of osteoblast differentiation.^23,24^ Both osteoblast differentiation and the expansion of osteoblast progenitors are essential for bone development and regeneration.^23,24^ Results confirmed early bone formation on the CAM model in FACaP1 and FACaP2, with a similar pattern to DBM positive control (Figure 4D). FA was used as a negative control, where a background RUNX-2 expression was seen, probably due to the transient expression of RUNX-2 in both endothelial cells and vascular smooth muscle cells of forming vessels.^25^ The expression of RUNX-2 in FACaP2 was greater than in FACaP1 and DBM. Previous results showed that the expression of osteopontin, as well as mineral deposition, were higher in FACaP2 compared with FACaP1 due to the high amount of CaP and the presence of OCP and/or HA phases in FACaP2, which induced a more marked differentiation of osteoprogenitor cells in FACaP2 compared with FACaP1.^17^ DBM does not contain a mineral phase, and osteogenic differentiation is induced by trace amounts of BMPs trapped in the DBM matrix.^26^ Therefore, we would expect a higher expression of the early osteogenic marker RUNX-2 in the FACaP2 scaffold compared to FACaP1 and DBM.

Altogether, our results show that our *ex ovo* CAM model serves as a biological bioreactor for the assessment of biocompatibility, angiogenesis, and early bone formation by biomaterials. Results also showed the angiogenic and bone forming capabilities of our developed FACaP scaffolds. The next part of our study investigated the possible mechanisms of this behavior for the FACaP materials.

Fourier-transform infrared spectroscopy (FTIR) spectra (Figure 5A) showed the surface changes in FACaP1 and FACaP2 following immersion in cell culture media for a week: phosphate group (PO_4_) burst peaks seen in FACaP1 samples at approximately 1050 cm^–1^ exhibited a high rate of reduction in the absorbance value at days 4 and 7. This suggests highly bioactive surface properties for FACaP1 compared to FACaP2, where surface changes were moderate. This was also confirmed via ion chromatography (IC) analysis, where a higher rate of calcium ion release was observed in FACaP1 following immersion in phosphate buffered saline (PBS) (Figure 5B).

**Figure 5:**
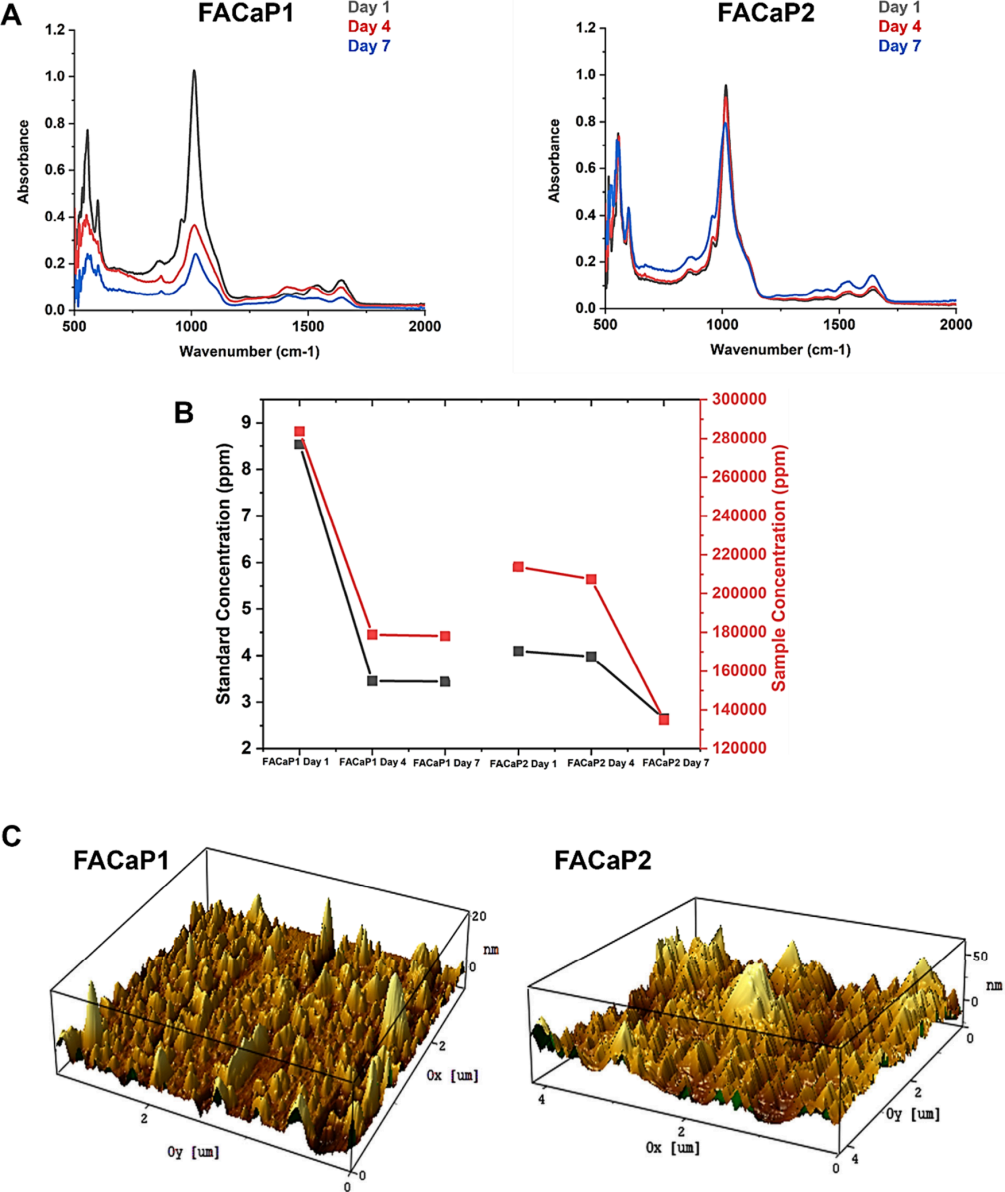
(A) FTIR spectra of FACaP1 and FACaP2. (B) IC presenting the rate of calcium ion release following immersion in PBS. (C) AFM photos of FACaP scaffolds.

Characterization of the surface properties of FACaP1 and FACaP2 was investigated by atomic force microscopy (AFM; Figure 5C). Both surfaces mainly displayed nanoscale fiber-like topographical features with interspaced nanospikes on top. FACaP2 exhibited a more random surface structure with higher nonuniform spikes of 50 nm, whereas FACaP1 showed a maximum of 20 nm with a more homogeneous structure. Upregulation of osteogenic markers on nanopatterned surfaces has been reported.^27,28^ Therefore, the role of nanotopographical features in FACaP1 and FACaP2 scaffolds must be highlighted in directing osteogenic differentiation and ultimately encouraging neo-bone formation.

In summary, we describe a novel, cost-effective, rapid, and simple method to assess bone regeneration in a nonsentient *in vivo* model based on an *ex ovo* CAM assay, thus refining and reducing the use of animal models in preclinical testing of biomaterials. Moreover, our novel FACaP scaffolds offer great potential as pro-angiogenic and osteogenic materials for bone tissue engineering due to their composition, nanotopography, and chemical properties.

## Supporting Information Available

Additional experimental details, materials, and methods (PDF)
