# Supplementary material for: Ex Ovo Chorioallantoic Membrane Assay as a Model of Bone Formation by Biomaterials
Source: ACS Macro Lett. 2024 Sep 26;13(10):1362–8. doi: 10.1021/acsmacrolett.4c00343 (PMC11483936; doi:10.1021/acsmacrolett.4c00343)
Supplement: Supplementary file 1 — mz4c00343_si_001.pdf [file mz4c00343_si_001.pdf]

Supporting Information

Ex ovo chorioallantoic membrane (CAM) assay as a model of bone formation by biomaterials

Nazanin Owji[1,2,3], Nupur Kohli[1,4,5], Oliver G Frost[1], Prasad Sawadkar[1], Martyn Snow[6], Jonathan C Knowles[2], Elena García-Gareta[1,2,7,8]

[1]Regenerative Biomaterials Research Group, The RAFT Institute & The Griffin Institute, Northwick Park and Saint Mark's Hospitals, Harrow HA1 3UJ, United Kingdom.

[2]Division of Biomaterials and Tissue Engineering, Eastman Dental Institute, University College London, London NW3 2QG, United Kingdom.

[3]Department of Biochemical Engineering, University College London, London WC1E 6BT, United Kingdom.

[4]Department of Biomedical Engineering, Khalifa University of Science and Technology, Abu Dhabi 127788, UAE.

[5]Healthcare Engineering Innovation Center, Khalifa University of Science and Technology, Abu Dhabi 127788, UAE.

[6]Royal Orthopaedic Hospital NHS Foundation Trust, Birmingham B31 2AP, United Kingdom.

[7]Multiscale in Mechanical & Biological Engineering Research Group, Aragon Institute of Engineering Research (I3A), University of Zaragoza, Zaragoza 50018, Aragon, Spain.

[8]Aragon Institute of Healthcare Research (IIS Aragon), Miguel Servet University Hospital, Zaragoza 50009, Aragon, Spain.

Corresponding author:

Elena García-Gareta
Aragon Institute of Engineering Research (I3A)
University of Zaragoza
Zaragoza 50018
Aragon
Spain
garciage@unizar.es

Materials and Methods

Biomaterials

Biomaterials used in this study can be seen on Table 1 and Figure 1 of the article. Demineralised bone matrix (DBM) is clinically available and was supplied by NHS-BT (Birmingham, UK). The fibrin/alginate (FA) material has been developed in our laboratory [1,2]. Briefly, the manufacturing process involves stirring reagents until they form a white foam; the foam is then casted onto a mould to solidify for one hour at 37°C. Subsequently, chemical crosslinking is carried out using 0.2% vol/vol glutaraldehyde (SigmaAldrich, UK) in an 80% ethanol/20% MES [2-(N-morpholino ethanesulfonic acid (69889, Sigma UK), 0.1M, pH=7.4] buffer for four hours at room temperature. Finally, the materials are rinsed with 0.1% wt/vol sodium borohydride (452882, Sigma-Aldrich, UK) in diH_2O, followed by a rinse with diH_2O, both at room temperature, and lyophilised (Virtis Genesis Freeze Dryer, Biopharma, UK).

FACaP composites were prepared by immersing the FA material in 5× concentrated simulated body fluid (SBF) solutions resulting in two prototypes (all solutions were filtered with a 0.22µm PES membrane before immersion) [3].

To prepare the SBF-1 solution, the following components were combined in 0.4 liters of distilled water: 0.695 grams of $CaCl_2$, 0.760 grams of $MgCl_2.6H_2O$, 0.880 grams of $NaHCO_3$, and 0.570 grams of $K_2HPO_4.3H_2O$. The pH was then brought to 6.0 using 1M HCl. Subsequently, 0.560 grams of KCl and 20.135 grams of NaCl were added to the solution. Finally, the pH was adjusted to 6.5 using 1M NaOH.

SBF-2 was prepared by mixing 20.135 grams of NaCl, 0.695 grams of $CaCl_2$, and 0.570 grams of $K_2HPO_4.3H_2O$ in 0.4 liters of distilled water. The pH was adjusted to 6.0 using 1M HCl.

FA materials were immersed in SBF-1 and stirred continuously at 150 rpm at 37°C for 24 hours. The materials were then rinsed with distilled water using an ultrasonic water cleaner for 60 seconds, frozen at -80°C, and subsequently subjected to lyophilisation (FACaP1). To produce FACaP2, the dried FACaP1 were immersed in SBF-2 and stirred at 60 rpm at 37°C for 48 hours. The materials were then washed, frozen, and lyophilised.

Scanning electron microscopy (SEM) of biomaterials

Biomaterials were Carbon coated before observation under SEM at 5 or 10 kV (Inspect F, FEI Company, The Netherlands).

CAM assay

The CAM assay employed in this study was detailed in Kohli *et al*. in 2020 [4]. Ethical approval was not required for this research, in accordance with the guidelines established by the Institutional Animal Care and Use Committee (IACUC) and the NIH (USA). These guidelines specify that a chick embryo at an age prior to reaching the 14th day of its gestation period would not experience pain, allowing its use in experiments without ethical restrictions or the need for prior protocol approval. To

summarise: a glass culture setup, as shown in Figure 3 of the article, was used. Pyrex glass containers with an approximate diameter of 8 cm were sterilised via autoclaving and filled up to about three-quarters with sterile water. A pre-sterilised cling film layer, prepared using 70% industrial methylated spirit (IMS) and dried, was placed inside the containers, ensuring that the cling film's bottom made contact with the water. Next, 500 µL of an antibiotic/antimycotic (AM) solution (Sigma, Dorset, UK) was pipetted onto the cling film, which was kept in place with rubber bands.

Fertile chicken eggs were placed in an egg incubator with automatic rotation for three days at 38°C and 45%-50% humidity. On the third day (embryonic day (ED) 3), the eggs were cleaned with cytosol, cracked open, and their contents transferred to the glass culture setup. Viability of the embryo was determined by observing the presence of a beating heart. To prevent contamination from the eggshells, 500µL of AM solution was gently pipetted onto the albumen. The glass containers were covered with a Petri dish and then incubated at 38°C and 80%-90% humidity for six days to facilitate embryo and CAM development (Figure 3). On the ninth day (ED9), up to six biomaterials were implanted onto the CAM, as illustrated in Figure 3. The ex ovo cultures were further incubated for three more days. On the twelfth day (ED12), embryos were euthanised in accordance with British Home Office regulations by freezing at -20°C for approximately 15 minutes. To prevent bleeding of the CAM after excision, 5mL of 4% paraformaldehyde (PFA) was applied to cover the CAM. The scaffolds were then carefully dissected from the CAM with a 5mm margin of the CAM tissue excised along with the material and stored in 4% PFA. Images were captured by inverting the biomaterials to observe the infiltration of blood vessels using a GT vision stereo microscope (GXM-XTL3T101).

Immunohistochemistry and confocal microscopy

Scaffolds in 4% PFA were embedded in paraffin wax and sectioned into 4-µm-thick slices. The sections were washed with PBS and incubated overnight at 4 °C with RUNX-2 or alpha smooth muscle actin (Abcam, UK) antibodies. After rinsing with PBS, the sections were incubated in AlexaFluor488-conjugated secondary antibody (Cell Signalling, USA) in a humidified chamber for 1 hour at room temperature. The nucleus was stained with DAPI. The fluorescence images were acquired by confocal laser scanning microscopy (Zeiss LSM 510, Carl Zeiss, Germany).

Fourier transform infrared spectroscopy (FTIR)

FTIR was used to assess the molecular functional groups in FACaP1&2 following incubation in cell culture media: the scaffolds were placed in contact with Attenuated Total Reflectance accessory (Golden Gate ATR, Specac, UK). The Spectrum software version 5.0.1, provided by Perkin-Elmer in the UK, was employed to identify the peak intensities of each chemical group. The wavenumber range used for the analysis was set between 500 and 4000 cm^{-1}, and the resolution was set at 4 cm^{-1}.

Ion Chromatography (IC)

The calcium ion release measurements were conducted using an Ion Chromatography system (ICS 2500). To prepare the samples, 550 µl from each sample was pipetted into appropriately labelled plastic vials. The vials were then placed in a designated

metal vials tray, which was subsequently inserted into the machine to detect ion release. For calibration purposes, standard solutions were created in five different dilutions, including 0 ppm, 1 ppm, 5 ppm, 25 ppm, and 50 ppm. A calibration curve was established based on the readings obtained from standard samples.

Atomic Force Microscopy (AFM)

Height and peak force error images were captured using a Bruker Dimension Icon microscope equipped with a Nanoscope V controller (Bruker UK Ltd, Santa Barbara, CA, United States). The microscope operated in peak force tapping mode with ScanAsyst Air cantilevers (with a nominal length of 115 μm, nominal width of 25 μm, and nominal spring constants of 0.4 N m^{-1}). The images were acquired at a resolution of 512 × 512 pixels, with a scan rate of 2 Hz and a peak force frequency of 2 kHz.

Data and statistical analysis

GraphPAD Prism 8.0.1 software was used. A one-way analysis of variance (ANOVA) was performed to compare groups. A p value below 0.05 was considered a significant result.

References

[1] Sharma V, Patel N, Kohli N, Ravindran N, Hook L, Mason C, et al. Viscoelastic, physical, and bio-degradable properties of dermal scaffolds and related cell behaviour. Biomedical Materials (Bristol) 2016;11. https://doi.org/10.1088/1748-6041/11/5/055001.

[2] Levin A, Sharma V, Hook L, García-Gareta E. The importance of factorial design in tissue engineering and biomaterials science: Optimisation of cell seeding efficiency on dermal scaffolds as a case study. J Tissue Eng 2018;9. https://doi.org/10.1177/2041731418781696.

[3] Kohli N, Sharma V, Orera A, Sawadkar P, Owji N, Frost OG, et al. Pro-angiogenic and osteogenic composite scaffolds of fibrin, alginate and calcium phosphate for bone tissue engineering. J Tissue Eng 2021;12. https://doi.org/10.1177/20417314211005610.

[4] Kohli N, Sawadkar P, Ho S, Sharma V, Snow M, Powell S, et al. Pre-screening the intrinsic angiogenic capacity of biomaterials in an optimised ex ovo chorioallantoic membrane model. J Tissue Eng 2020;11. https://doi.org/10.1177/2041731420901621.
